# A Review of *Toxoplasma gondii* in Animals in Greece: A FoodBorne Pathogen of Public Health Importance

**DOI:** 10.3390/ani13152530

**Published:** 2023-08-05

**Authors:** Isaia Symeonidou, Georgios Sioutas, Thomai Lazou, Athanasios I. Gelasakis, Elias Papadopoulos

**Affiliations:** 1 Laboratory of Parasitology and Parasitic Diseases, School of Veterinary Medicine, Faculty of Health Sciences, Aristotle University of Thessaloniki, 54124 Thessaloniki, Greece; isaia@vet.auth.gr (I.S.); gsioutas@vet.auth.gr (G.S.); 2 Laboratory of Hygiene of Foods of Animal Origin—Veterinary Public Health, School of Veterinary Medicine, Faculty of Health Sciences, Aristotle University of Thessaloniki, 54124 Thessaloniki, Greece; tlazou@vet.auth.gr; 3 Laboratory of Anatomy and Physiology of Farm Animals, Department of Animal Science, School of Animal Biosciences, Agricultural University of Athens, 11855 Athens, Greece; gelasakis@aua.gr

**Keywords:** *Toxoplasma gondii*, zoonotic parasite, prevalence, transmission, control measures, meat-producing animals, food safety, public health, abattoirs, foodborne pathogen

## Abstract

**Simple Summary:**

*Toxoplasma gondii* is a parasite that can infect humans and animals, mainly through meat consumption. It is also the second most important pathogen transmitted with food in Europe. However, detecting the presence of *T. gondii* in animal meat differs on a country basis since there are no mandatory controls along the food chain in the European Union. Underreporting of cases is still a problem in many countries like Greece. The current review examines the prevalence of *T. gondii* in animals in Greece and identifies the risks associated with meat transmission. Certain animals like sows, wild boars, hares, equines, and cats had lower levels of infection, while sheep and goats generally had higher levels compared to other European countries and to the global averages. The level of infection in chickens was similar between Greece and Europe, while there was high variation in cattle studies, with no data regarding dairy products. Until now, Greece has not implemented a comprehensive system to ensure meat safety, particularly regarding *T. gondii*. This review highlights the preventive measures that the state should implement to ensure food safety and protect public health, as well as the various control measures that should be adopted by consumers to reduce the infection risk.

**Abstract:**

*Toxoplasma gondii* is a zoonotic protozoon with a complex life cycle and the second most important foodborne pathogen in Europe. Surveillance of toxoplasmosis is based on national considerations since there are no mandatory controls along the food chain in the European Union, and underreporting of meat is still a problem in many countries like Greece. The current review provides an overview of *T. gondii* prevalence, associated risk factors, and surveillance in animals in Greece, focusing on the transmission role of meat and highlighting the control measures that should be adopted by consumers. Sows, wild boars, hares, equines, and cats had lower, while sheep and goats generally had higher seroprevalence than their respective pooled European and global values. Seroprevalence in chickens was similar between Greece and Europe, while there was high variation in cattle studies, with no data regarding dairy products. Though a comprehensive meat safety assurance system is the most effective approach to control the principal biological hazards associated with meat, such as *T. gondii*, the prerequisite risk categorisation of farms and abattoirs based on EFSA’s proposed harmonised epidemiological indicators has not materialised as yet in Greece. Therefore, comprehensive control strategies are still required to ensure food safety and safeguard public health.

## 1. Introduction

Among foodborne pathogens, parasites have been neglected primarily due to their complex life cycles, prolonged incubation period, several transmission routes, and chronic impact on hosts [1]. However, several zoonotic foodborne parasites (FBP) are today considered emerging threats and have been increasingly recognised as being responsible for considerable disease burdens worldwide [2] based on multicriteria decision analyses and estimations [3]. In this context, the second-highest-ranked FBP in Europe is the zoonotic protozoon *Toxoplasma gondii* (*T. gondii*) [4].

This obligate intracellular coccidian parasite has an indirect life cycle, with the sexual reproduction occurring only in the small intestine of Felidae (definitive hosts) and asexual multiplication taking place extra-intestinally into the tissues (tissue cysts) of all warm-blooded animals, including humans (intermediate hosts). There are three different infectious stages: sporozoites in oocysts, tachyzoites, usually in secretions, and bradyzoites in tissue cysts [5]. When hosts get infected, tachyzoites quickly proliferate inside different cells [6]. Consequently, tachyzoites form cysts in different tissues and develop into bradyzoites. These tissue cysts survive throughout the host’s lifetime and can infect any human or animal that consumes them. Thus, the consumption of undercooked or raw meat from infected meat-producing animals may pose a risk to public health [6,7]. Oocysts are shed with the faeces of infected felids, particularly kittens, sporulate and may contaminate food, fresh produce, shellfish, and water leading to human infection following consumption [7,8,9]. In addition, humans acquire *T. gondii* by ingesting undercooked meat containing viable tissue cysts or unpasteurised milk and dairy products containing tachyzoites [10,11]. Toxoplasmosis is also an occupational disease for hunters, butchers, and slaughterhouse workers who may become infected during evisceration [12,13,14,15,16].

Foodborne transmission is considered the primary mode of human infection with *T. gondii*. A European multicenter case-control study depicted that 30–63% of *T. gondii* infections in humans could be attributed to meat consumption, including cured meat and wildlife meat, i.e., deer [12]. In the same frame, available data indicated that foodborne transmission accounts for 40–60% of human toxoplasmosis, and the most commonly implicated food sources are the meat of ruminants and pork, as well as vegetables [11,17]. It should be noted that consumers prefer “ready-to-eat” products and favour meat from animals raised in organic farms, i.e., with access to outdoor grazing. On top of that, the tendency to consume rare or raw meat not previously frozen may also increase the risk of ingesting infective *T. gondii* tissue cysts [7].

Regarding clinical symptoms, *T. gondii* is considered the most prevalent parasitic zoonotic infection globally [7,8], leading to various diseases in humans and animals. Approximately one-third of the global human population is estimated to be chronically infected with *T. gondii* [18]. Acquired human toxoplasmosis is typically subclinical, while mild and unspecific symptoms sometimes occur. Long-term consequences, such as ocular symptoms, may exhibit years later [11]. The neurological form of the disease in humans, cerebral toxoplasmosis, has also been associated with schizophrenia, psychiatric and bipolar disorders, among other conditions [18]. Furthermore, acquired toxoplasmosis can be fatal for immunosuppressed individuals and is ranked as the leading cause of death for this population group [19,20]. In particular, the World Health Organization (WHO) has ranked acquired toxoplasmosis and ascariosis as the parasitic diseases with the largest total number of symptomatic cases and, most interestingly, symptomatic cases that are attributed to contaminated food [21]. In the congenital form of the disease, infected children can develop blindness and mental retardation, and infection can even be fatal for the fetus during pregnancy. Studies have demonstrated that the global estimated incidence of congenital toxoplasmosis in humans is 190,100 new cases per year [19,22]. Both congenital and acquired toxoplasmosis have an elevated public health impact [11,17,21]. Toxoplasmosis is the fourth most common cause of hospitalisation and the third leading cause of death among foodborne diseases [6]. Conclusively, *T. gondii* is an important foodborne pathogen that ranked high in Europe based on the multicriteria decision analyses (MCDA) methods [3] and disability-adjusted life-years (DALY) estimates for disease [2].

The current review provides a detailed overview of *T. gondii* prevalence and surveillance in animals in Greece. Moreover, it highlights the central role of different types of meats in *T. gondii* transmission and the primary prevention strategies and measures to limit foodborne transmission.

## 2. Surveillance across Europe

In humans, congenital toxoplasmosis is notifiable in many European countries. Screening pregnant women is mandatory in some countries (Belgium, France, Slovakia, Croatia, Italy, Poland, Serbia, and Slovenia), while it remains voluntary in others (Bulgaria, Hungary, Czech Republic, and Germany) [23]. Population-based serosurveillance studies have been reported from Australia, Belgium, Germany, Spain, France, Iceland, the Netherlands, Norway, Portugal, and Sweden [24,25,26,27]. Data from these studies in the Netherlands have demonstrated that toxoplasmosis has one of the highest disease burdens among foodborne diseases [2]. In most European countries, passive surveillance of human clinical cases of both hospitalised and other patients exists, but whether these cases are systematically reported is unclear. Nonetheless, underreporting is still a problem in many countries and is attributed mainly to the lack of concise rules for recording. Therefore, more effort is required to improve the assessment of the disease burden and thus prioritise adequate control measures.

As regards livestock, surveillance of toxoplasmosis is voluntarily implemented on a national level since there are no mandatory controls and no regular official recordings regarding *T. gondii* along the food chain in the European Union (EU) [11]. Specifically, animal toxoplasmosis is notifiable in 14 European countries, such as Belgium, Czechia, Germany, Finland, France, Iceland, Ireland, Liechtenstein, Poland, Slovenia, Latvia, North Macedonia, Serbia, and the Netherlands [4]. However, the obtained data are considered of rather limited value due to various factors such as small sample sizes, non-harmonised sampling schemes, different diagnostic methods, and lack of animal-related information (e.g., age and rearing system) [4,15]. All of the above render impossible the accurate estimation of the prevalence of *T. gondii* infections in livestock at the EU level [28,29,30,31]. Passive surveillance, i.e., recording animals with compatible clinical signs, such as abortions in small ruminants, is applied in some countries. No obligatory serological monitoring of incoming animals for slaughter is in place in the EU, and optional active surveillance at the abattoir level is carried out inconsistently by serology and molecular methods. Therefore, it cannot substitute routine inspection and ensure meat safety. It should be noted that post-mortem macroscopic examination (visual inspection) of infected meat is unsuitable for *T. gondii* detection since tissue cysts are too small (100 μm) to identify without using microscopy [6]. In fact, *T. gondii* meat detection and identification is applied mainly during certain research projects and outbreak investigations of congenital toxoplasmosis. Therefore, surveillance in the EU is relatively inadequate for meat-producing animals. This gap reinforces the necessity of applying risk-based surveillance systems in livestock based on risk assessment surveys similar to the ones already available in the Netherlands and Italy [11] to prevent human meat-borne infections and reduce the disease burden.

## 3. Diagnostic Approaches

The European Food Safety Authority (EFSA) BIOHAZ Panel has highlighted a paucity of robust and validated diagnostic tools for *T. gondii* that can be utilised across different types of foodstuffs, including meat samples [11]. This paucity along the parasite’s heterogeneous life cycle constitutes a serious impediment to source attribution studies.

Many studies assess the seroprevalence of *T. gondii* [32]. One should keep in mind that each study presents a unique approach toward the seroprevalence estimation of the parasite in various animal species and cannot be directly compared with other studies since a vast number of factors such as different diagnostic methods employed, location, population size, animals’ age, breed, gender, weight, farming system, presence of cats and regional climatic conditions influence the outcome [33,34]. Moreover, serology can only be used to estimate the risk of human infection if a correlation exists between seroprevalence and tissue cysts in meat. This correlation has been studied for the main livestock species in an extensive review, and it was demonstrated that the likelihood of detecting parasites in seropositive animals was highest in pigs (58.8%), followed by chickens (53.4%), sheep and goats (39.4% and 35.0%, respectively), and was lowest in horses (8.8–13.8%) and cattle (3.6%). Therefore, the seroprevalence can be utilised to estimate the public health risk of meat-borne toxoplasmosis only as regards these livestock species, but it is not applicable in the case of cattle and horses, in which similar detection rates of the parasite have been reported between seropositive and seronegative animals [35]. Another disadvantage of serology is that there are serological non-responders, i.e., seronegative animals with tissue cysts. This phenomenon has been reported in pigs (4.9%), sheep and goats (1.8% and 2.0%, respectively), chickens (1.8%), cattle (2.4%), and horses (2.4–32.0%). As a result, a seronegative animal may produce *T. gondii*-infected meat, and serology cannot be employed for individual carcass control [35].

From a public health perspective, this lack of data on the prevalence of *T. gondii* tissue cysts in cattle and horses remains a crucial gap as beef is a major meat source in many countries in Europe and horse meat in some, such as France and Italy. In this frame, a quantitative risk assessment for meat-borne toxoplasmosis was performed in the Netherlands, and it revealed that beef (rather than pork or mutton) contributed to 67% of the predicted human cases [36]. Moreover, since beef and horse meat are more frequently consumed undercooked or raw than pork or poultry, usually eaten well-cooked [35], this information is essential to accurately reflect the public health risk involved. Several serological studies have been published in Europe (reviewed by Tenter et al.) [32], and seroprevalence ranked from 4% to 92% in sheep, 2% to 92% in cattle, 4% to 77% in goats, 0% to 64% in pigs, and 0 to 53% in horses depending on the husbandry system [37]. Relatively high seroprevalence rates have been observed in sheep and goats in Mediterranean countries, thus pinpointing mutton as an essential meat source of *T. gondii* infection for consumers [37].

## 4. Studies on *T. gondii* in Animals and Their Products in Greece

Different cross-sectional studies, mainly serological ones, and case reports are available on *T. gondii* in both domestic and wild animals in Greece, namely in domestic swine and wild boars [38,39,40], sheep [41,42,43,44,45,46,47,48,49,50], goats [41,42,43,45,46,47,48,51], cattle [52,53,54], birds [55,56,57], hares [58], equines [59], cats [60,61,62], wildcats [63], and in one camel [64] (Table 1). Some of these animal species, i.e., cats, chickens, or even hares, can be used as sentinels for human infection in specific regions, and their seropositivity can prove helpful in assessing the environmental contamination with oocysts [7,14,57,60]. The prevalence rates discovered in the examined research are compared with worldwide and EU prevalence rates obtained by systematic reviews and meta-analysis studies.

### 4.1. Pigs and Wild Boars

The occurrence of *T. gondii* in pigs has been addressed in two studies in Greece. Compared to the worldwide pooled seropositivity in sows (19%) as well as the average European seropositivity (13%) [33], sows in Greece exhibited lower seropositivity (4.3% and 4.4%) [38,39]. Generally, pork originating from organic farms is more frequently infected than conventional farms [65]. In Greece, sows in mountainous areas and farms with low biosecurity measures had a significantly higher risk of infection than those in lowland areas and farms with high biosecurity measures [39]. In addition, sows not vaccinated against porcine circovirus 2 had higher seropositivity rates. This association most likely resulted from inadequate practices in farms with unvaccinated sows, increasing the infection risk with *T. gondii* [38]. There is only one relevant study conducted on wild boars in Greece, which documented a seroprevalence of 5.2%, a rate also lower than the global pooled seropositivity (23%) and the respective European one (26%) in wild boars [7,13]. Possible risk factors for wild boars include dense populations of boars and confined geographic regions [40].

Nonetheless, swine seroprevalence is not always correlated to the existence of *T. gondii* bradyzoite tissue cysts in pork meat [6]. Similarly, detecting antibodies or cysts in slaughterhouse samples does not indicate human infection risk due to storing and processing procedures of pork meat after slaughter that can destroy *T. gondii* cysts [7]. As is the case in Greece, in many countries, the routine cooking of pork combined with the low seroprevalence has significantly reduced the risk of pig-to-human transmission of *T. gondii* [32]. In this context, a meta-analysis assessing the risk of different foods in human toxoplasmosis did not regard consuming undercooked or raw pork as a significant risk factor [66]. Despite this risk reduction, in the USA, pork meat is still considered a significant threat to human *T. gondii* infection [67]. Consumers should know that pigs’ brains, lungs, hearts, and tongues are the most commonly infected organs with *T. gondii* and have a higher parasitic burden than other organs [68,69,70].

### 4.2. Sheep and Meat Thereof

*Toxoplasma gondii* seroprevalence in Greek sheep has been extensively studied throughout the years [41,42,43,44,45,46,47,48,49,50]. Estimations ranged from 23% in Crete in 1995 [48] to 90% in Trikala in 2019 [44], with most studies finding seropositivity of around 50% and an upward trend throughout the years, as seen in Table 1. Compared to the global pooled seroprevalence in sheep at 33.86% and the pooled European seroprevalence at 41.01% [71], it becomes evident that Greece has higher seroprevalence and is endemic for ovine toxoplasmosis.

Furthermore, toxoplasmosis can be one of the leading abortion causes in sheep. The seropositivity of *T. gondii* in abortive sheep has been calculated at 52.1% [46], 60.9% [43], and 49.8% [47] in different studies in Greece. These seropositivity rates are similar to the global pooled seropositivity of *T. gondii* in abortive sheep calculated at 56% [34]. In one case report, 60% of pregnant ewes aborted, and *T. gondii* was diagnosed as the causative agent [50]. However, other studies demonstrated no association between *T. gondii* seroprevalence and abortions in ewes [41,43]. This discrepancy among studies is expected, considering that abortion rates are typically much higher in naïve ewes that acquire the infection for the first time during pregnancy. In contrast, in sheep exposed to *T. gondii* before pregnancy, immunocompetence establishes, and abortion rates are usually much lower [34,50].

Common risk factors for ovine toxoplasmosis include a herd larger than 300 sheep, most likely increasing infection risk due to overcrowding and increased exposure to infection sources [45]. Sheep co-grazing with other herds may also have an increased probability of infection because it is associated with poor biosecurity measures [45]. Sheep reared under intensive or semi-intensive management schemes, offered feed concentrate, and water from public systems might have a higher risk of infection [41]. The elevated infection risk in these groups may be attributed to a higher stocking density in intensive systems and more intermediate hosts, such as rodents in feed warehouses, compared to extensive systems, while the “water from public systems” might be a confounder [41]. In regards to climatic conditions, high temperatures, low rainfalls, and low altitudes were also associated with increased *T. gondii* seropositivity in one study, probably deriving from oocyst susceptibility to different weather conditions [45]. In contrast, another study found no difference in ovine seroprevalence between coastal and mountainous regions [41]. Higher seroprevalence has also been documented in sheep living in urban and agricultural/forest areas compared to savanna areas, possibly associated with the presence of infected client-owned or stray cats that excrete oocysts in those former areas [45]. Ewes had a significantly higher seroprevalence than male sheep in one study [43], most likely because male sheep have a more robust immune response against the parasite due to hormonal differences [72,73,74]. This finding agrees with a recent meta-analysis on sheep *T. gondii* seroprevalence and associated risk factors [71]. Regarding rams, sexual transmission is possible, and an experimental study demonstrated that *T. gondii* reduces the quality parameters of sperm (viability, motility, velocity), while sulphadimidine treatment did not revert the sperm cell morphological abnormalities [75]. Concerning age, sheep older than four years had higher seroprevalence than younger sheep, most likely due to increased exposure to *T. gondii* and not because old sheep are more susceptible [7,43,71,76]. It is worth noting that cats were present in all farms in one of these studies and had free access to the feedstuff [43]. As definitive hosts, cats can be a source of infection by expelling oocysts with their faeces on the sheep’s grazing pasture or feed [32,42,71]. However, the presence of cats on the farm level is not always a significant risk factor [41].

Sheep meat is one of the most commonly infected foods regarding human toxoplasmosis [32]. A meta-analysis investigating the prevalence of *T. gondii* in meat from different animals identified sheep meat as the most infected animal meat, with a global mean prevalence of 14.7%, even surpassing pork (12.3%) [65]. Another meta-analysis classified the consumption of sheep meat as the most crucial risk factor for human foodborne infection with *T. gondii* [66]. Given the high seroprevalence of *T. gondii* in sheep in Greece and its growing trend, awareness should be raised in farmers to employ strict biosecurity measures based on risk factor analysis to prevent their animals from infection. Towards this end, screening methods for *T. gondii,* both at the farm and slaughterhouse level, should be put in effect, and consumers should practice standard hygiene measures and cook mutton thoroughly to reduce infection risk, particularly in the case of immunocompromised individuals [77].

### 4.3. Goats and Meat Thereof

The seropositivity of *T. gondii* in goats has been researched alongside sheep seroprevalence, displaying fluctuation through the years and estimated at 14% (1995) [48], 50.4% (2002) [46], 17.9% (2007) [43], 30.7% (2012) [41], 16.8% (2013) [45], and 61.3% (2013) [42]. Comparatively, the worldwide pooled seroprevalence of *T. gondii* in goats is in the middle at 31.7% and the European value at 38.8%, exhibiting high heterogenicity among the tested regions [71]. In Greece, three studies found that sheep had a significantly higher seroprevalence than goats [41,43,48], but one study reached the opposite conclusion [42]. At a global level, sheep have a higher seroprevalence than goats, but the difference is not statistically significant [71]. Sheep have a higher infection risk than goats due to feeding habits; goats are browsers and feed from plant leaves high from the ground, while sheep are grazers and feed from the vegetation on the ground that is more commonly contaminated with *T. gondii* oocysts [42]. Although there is a difference between the two animal species, in Greece, both sheep and goats are kept indoors and fed similar food during the colder months. This practice reduces discrepancies in their feeding habits and equalizes the infection risk [42]. However, it is important to note that further research or in-depth studies may be necessary to fully understand the situation in Greek small ruminants. Sheep might additionally have a genetic predisposition to toxoplasmosis compared to goats [41].

In goats, transplacental transmission leading to abortion can also occur if the mother has been infected before pregnancy when tissue cysts get re-activated, which is uncommon in sheep that typically have more robust immune protection [34]. Consequently, goats may abort in multiple breeding periods, which is relatively rare in sheep that usually only abort once [7]. In different studies, the seroprevalence of *T. gondii* in abortive goats was calculated at 47.9% (2002) [46], 14.3% (2007) [43], and 29.9% (2009) [47]. However, *T. gondii* seropositivity is not always significantly different between abortive and non-abortive goats [41,43]. Compared to Greece, the global pooled seroprevalence of *T. gondii* in abortive goats is higher, estimated at 50% [34], indicating that *T. gondii* is present as a cause of reproductive failure in Greek goats but not as common as in other countries. Nevertheless, this parasite can cause massive abortions in goats, and in a reported case of natural toxoplasmosis in three dairy goat herds, abortion rates reached as high as 78.5% without treatment [51].

Concerning risk factors, goats in intensive or semi-intensive farms and those in large herds (>300 animals) have a higher infection risk [41,45]. Goats are more crowded in intensive farms, and large herds are in closer contact with infection sources like young cats and their faeces [41,45,47]. In like manner, providing goats feed concentrate may increase infection risk because cats with free access to the feed can expel oocysts with their faeces and contaminate them [41]. Furthermore, seropositivity in goats increases with age, like in sheep, because goats have antibodies for many years after they come in contact with the parasite, and as they age, their chances of exposure to *T. gondii* increase [43]. Weather conditions such as high temperatures, low rainfalls, and farm altitude can also potentially increase infection risk due to increased oocyst survival in these climates [45].

In goat meat, *T. gondii* predilection sites include the lungs, brain, and dorsal muscles, which also have a higher parasitic burden than other tissues [78]. Regardless of the tissue location, cysts have an unequal disposition in goats’ meat [7], which may present a higher risk of transmitting the parasite to humans than other meats like pork [39].

### 4.4. Cattle and Meat Thereof

Cattle are generally poor hosts to *T. gondii*, resistant to disease, and have low seropositivity compared to small ruminants [79]. Laboratory studies have demonstrated that although cattle can acquire the parasite just as easily as small ruminants, *T. gondii* does not survive long in their tissues, and the number of cysts decreases close to zero within a few days [80]. Consequently, cattle become seronegative after a while, so seroprevalence studies cannot be used as an indication for cattle harbouring cysts with bradyzoites [81]. In Greece, three previous studies calculated cattle seropositivity at 39.7% (1992) [53], 20.0% (2005) [54], and 8.1% (2020) [52], indicating that the pathogen is present in cattle farms, irrespective of the farm management system [54]. However, none of the three studies examined possible cross-reactivity between *Toxoplasma* spp., *Neospora* spp., and possibly other cyst-forming coccidia (e.g., *Besnoitia* spp., *Sarcocystis* spp.) that are relevant for cattle [82]. Despite this, a decline in seroprevalence rate was observed through the years, just like in the rest of the world [83], although each study was conducted in a different region. Still, the seroprevalence of *T. gondii* in Greek cattle was higher in two studies than the global pooled seroprevalence in cattle at 16.9% [83]. Nonetheless, reproductive failures and calf mortality due to *T. gondii* infections are very rare in cattle [34,54], with *Neospora caninum* infections being much more common [34].

No specific risk factors for cattle toxoplasmosis in Greece have been investigated before, but in two studies, adult cattle had higher seropositivity than young ones, suggesting that the risk of infection increases as cattle get older [52,54]. Again, this increase can be attributed to increased exposure to *T. gondii* infection sources, like oocysts in the environment [52].

Consuming raw or undercooked beef has been established as a significant risk factor for human toxoplasmosis [34,66,84]. This finding contradicts the knowledge that cattle are typically considered unsuitable hosts for *T. gondii,* and beef is rarely infected with the protozoon [66]. Nonetheless, consumers’ habits of eating beef, typically raw or undercooked, compared with other types of meats that are preferred cooked (i.e., pork) have led to beef being a significant infection source for human toxoplasmosis [66]. The impact of beef on human toxoplasmosis is also enhanced by the large quantities of beef consumed each year compared to other types of meat [85], as well as by the fact that beef constitutes a major meat source in several European nations. In fact, as mentioned earlier, in one study in the Netherlands, beef caused more than 67% of *T. gondii* meat-borne infections [36].

### 4.5. Birds and Poultry Products

Birds have three prominent roles in the epidemiology of *T. gondii*. Firstly, they are common prey for cats in urban and rural environments; secondly, domestic (i.e., chickens) or wild birds (i.e., game meat) are consumed by humans, and lastly, birds can cover large distances transporting *T. gondii* to new previously uncontaminated locations [55].

As regards seropositivity in Greek birds, in the single study conducted in chickens, the overall seroprevalence of *T. gondii* was 9.4%, with backyard chickens exhibiting much higher seropositivity at 41.2%, layers at 2.8%, while all broilers were negative [57]. Only one broiler chicken was positive after a PCR examination [57]. These findings align with other studies on *T. gondii* in chickens that showed a high seroprevalence in backyard chickens, a low seroprevalence in layers/caged chickens, and an almost zero seroprevalence in broilers [7]. In another study on domestic and urban (wild) pigeons in Northern Greece, seropositivity using ELISA was 5.8% and 0%, respectively [56], although a small number of wild pigeons was examined (n = 50). When examining woodcock populations from two different areas of the country with PCR, 4.7% of woodcocks were positive for *T. gondii*, indicating that wild birds harbour the parasite in Greece, too [55].

Significant risk factors for infection included chickens with outdoor access, meaning they grazed freely, and those that fed without an automatic feeder [57]. An explanation could be that free-range chickens eat transport hosts (i.e., earthworms) that mechanically carry *T. gondii* oocysts [57]. This result is consistent with other studies revealing a lower seroprevalence of *T. gondii* in caged chickens [7]. Surprisingly, the presence of cats was not a significant risk factor, indicating that chickens may not acquire the infection directly through the faeces of infected cats but through other sources [57]. There were no significant differences in seroprevalence among the regions examined [57]. Seroprevalence studies in chickens could benefit the food industry since chickens remain seropositive for a long time, and also, there is a good association between the existence of antibodies and the presence of *T. gondii* DNA in chickens [57]. Additionally, a strong positive correlation exists between antibody titers and parasitic burden in chickens [7].

In a similar pattern, retail chicken meat can be PCR positive for *T. gondii*, with the prevalence reaching up to 10% in some countries [86,87], even surpassing beef and pork in one case [88]. However, in some countries, chicken meat is typically sold frozen in retail stores, a practice that kills *T. gondii* tissue cysts [7]. In regards to other poultry products and *T. gondii*, consuming raw eggs was not considered a significant risk factor for foodborne human toxoplasmosis in a meta-analysis, despite a few old studies describing the detection of *T. gondii* in eggs [7,66].

### 4.6. Hares and Meat Thereof

There is only one available study that detected a 5.7% seroprevalence of *T. gondii* in hares in Greece [58]. This seroprevalence was on the lower end of *T. gondii* seropositivity in hares from other studies and countries, ranging from 0–21% [14]. Interestingly, no liver samples were positive for the protozoon with PCR [58], like in some other studies investigating the presence of *T. gondii* in different hare tissues [14]. Despite these results, PCR has successfully detected the parasite in some infected hares; thus, *T. gondii* tissue cysts could be present in the meat of seropositive hares [14]. Regardless of the low seroprevalence in Greece, the demand for hare meat is on the rise, posing a risk, especially for hunters that consume raw/undercooked game meat [7,14]. Rainfalls and the type of land hares lived on (i.e., forests and grasslands) were assessed as significant risk factors for *T. gondii* infection due to increased oocyst survivability and dense populations of wildcats/transport hosts, respectively [58].

### 4.7. Equines and Meat Thereof

In one study conducted on 770 equines (753 horses, 13 mules, and 7 ponies) carried out by Kouam et al. in 2010, the seroprevalence of IgG against *T. gondii* was 1.8% [59]. This seropositivity rate was relatively low compared to the global pooled equine seroprevalence at 11.3% [89]. Equines used in farms and equines in Thessaly and Peloponnese had a significantly higher seroprevalence than those used for racing or recreation and equines living in Attica, respectively [59]. Horses in farming probably acquired the parasite by ingesting oocysts from the environment (contaminated water or feed) or through the accidental ingestion of infected meat or offal [59]. Despite the low seroprevalence, *T. gondii* has been detected in more than 50% of horse meat using mouse bioassays in two surveys in Egypt and Brazil [65]. Consumption of horse meat is extremely rare in Greece and, thus, is not regarded as a vital source of human infection [59]. However, monitoring equine infection is necessary due to possible adulteration cases of beef with horse meat, like in the 2013 European scandal [90].

### 4.8. Cats

Felines are the only definitive hosts of *T. gondii* and excrete oocysts with their faeces [60]. However, cats typically shed oocysts 6–10 days after primary infection [91] and 4 days after re-infection [92]. Therefore only 0.4% of domestic cats shed oocysts whenever faeces are collected [93], and the prevalence of 0% [61] and 0.4% [62] of *T. gondii*-like oocysts in the faeces of cats examined in two previous studies in Greece are unremarkable. Concerning wild felids, 2.4% of them expel oocysts at any given moment globally [93], and this is in concordance with the prevalence of *T. gondii*-like oocysts in the faeces of wildcats in Greece (1.6% and 4.3%) [63]. It should be kept in mind that when conducting faecal studies for *T. gondii*, oocysts need to be further identified with molecular methods (i.e., PCR) because the feline coccidia *Hammondia hammondi*, *Besnoitia* spp., and *T. gondii* all share the same morphology and cannot be differentiated with microscopy [60,63].

The countrywide seroprevalence of *T. gondii* in cats in Greece was recently estimated to be 21.8% [60], which is lower compared to both the global pooled seroprevalence at 37.5% and the European seroprevalence at 45.3% [94]. Cats in the geographical region of Peloponnese displayed the highest seropositivity at 42.8% and, interestingly, previous studies also detected high *T. gondii* seroprevalence in sheep [43] and equines [59] in this specific area, indicating a potentially high environmental oocyst burden in Peloponnese [60].

Risk factor analyses identified cats in rural areas, cats that hunted, and cats with outdoor access as having a significantly higher probability of infection [60]. Rodents, the protozoon’s natural secondary host, and other small animals, birds, and transport hosts can harbour *T. gondii,* and cats that hunt can get infected when eating them [60]. Similarly, cats with outdoor access can hunt more often, and cats in rural areas come in contact with more intermediate hosts and oocysts in the environment [60]. In the binary logistic regression model, hunting in urban areas remained the only significant risk factor, indicating that most cats in Greece acquire the infection by ingesting bradyzoites in tissue cysts, the natural transmission of *T. gondii* for cats [60,95].

### 4.9. Dairy Products

Consumption of raw milk is also considered a risk factor and a possible foodborne transmission route for tachyzoites, which are the parasite stage likely to be shed in the milk of infected animals during lactation [11,96]. Although drinking milk is generally not a significant risk factor for *T. gondii* infection [66], explicitly drinking unpasteurised goat milk is [84]. Indeed, human toxoplasmosis cases have been attributed to the consumption of raw milk from infected goats [11,97]. Pasteurisation and low pH values are generally regarded as sufficient to inactivate tachyzoites. However, it has been evidenced that the latter survived for at least 60 minutes in gastric fluids mixed with various quantities of artificially spiked cow’s milk samples due to gastric pH value fluctuations [7,96]. In European countries, the prevalence of *T. gondii* in raw milk samples via PCR assays has been reported to range from 4% to 11% as regards sheep milk samples [98,99,100] and from 4% to 65% with reference to goat milk samples [99,101,102] whereas 16% of cow milk samples were found positive in one study [99]. However, data are scarce regarding the occurrence of *T. gondii* in raw milk from Greek farms. In an experimental study in Greece, *T. gondii* was detected in ovine and caprine milk samples until 28 days post-infection (p.i.) [103]. As in the case of raw milk, the consumption of related unpasteurised dairy products (e.g., whey, fresh cheese) represents another route for transmission to consumers, and *T. gondii* has been detected in cheese originating from unpasteurised goat milk [104]. In contrast, cheese from sheep milk is deemed safe for human consumption, probably due to the cheese-making process (pH, salt concentration) that deactivates any *T. gondii* in the milk [77].

## 5. Control Measures for Foodborne Toxoplasmosis

As stated before, *T. gondii*-relevant data in current food chain information are limited, and current meat inspection practices lack effectiveness in reducing human toxoplasmosis health risks attributed to meat consumption [29,30,31,105]. In addition, cross-contamination is a key aspect of HACCP and food safety management systems for controlling foodborne bacteria in the meat production chain but not an issue of concern in the case of intracellular parasites, such as *T. gondii*.

Given the above, consumers are responsible for employing appropriate food-handling practices [65]. Water deactivates tissue cysts, and thus, after preparing meat, hands should be properly cleansed with soapy water [7]. Kitchen utensils, knives, cutting boards, and countertops used to prepare meat should be washed with hot water and soap and cleaned frequently to avoid cross-contamination of food products [66]. Moreover, it is recommended that people only drink pasteurised milk (especially if it is from goats) and treated-filtered water. In particular, lake or river water should first be boiled [106]. It is worth mentioning that meat consumption per se or eating meat frequently is not associated with a higher risk of *T. gondii* infection [66]. Generally, consuming raw/undercooked [66] cured, smoked, locally produced, or age-dried meat [84] is unsafe and can significantly increase the risk of infection with *T. gondii*. Similarly, tasting the meat while it is being cooked should be avoided, unlike tasting seasonings, which are considered safe [66]. Mollusks and other sea animals should also be cooked before consumption [107].

Several options are available for killing tissue cysts containing bradyzoites in infected meat, and they are separated into three large categories: freezing, cooking, and alternative methods [108,109,110]. Freezing infected meat can kill tissue cysts if the temperature reaches −20 °C for 3 days minimum, while lower temperatures can inactivate cysts even quicker [6,109,110]. Heating is the most commonly used method for destroying *T. gondii* tissue cysts in meat, provided that the internal meat temperature is at least 67 °C for around a minute [6,110]. Consumers should be aware that the thickness and kind of the meat cut will affect the time required to kill tissue cysts [7]. Using a sheet of aluminium foil can aid in the even spread of heat across the meat [6]. Some alternative methods that have proven effective include meat irradiation at 75–100 krad and high-hydrostatic pressure processing at 340–400 MPa for at least 1 minute, but their high price and alteration of meat organoleptic characteristics (texture, colour) make them less preferable options [6,109,111,112].

On the other hand, microwaving is ineffective in destroying *T. gondii* tissue cysts in meat [6] due to asymmetrical heating [108]. In like manner, salting or smoking are unreliable methods for deactivating *T. gondii* tissue cysts in meat such as pork [32] since some tissue cysts may survive curing [6]. Furthermore, the time needed for bradyzoite destruction by curing meat is far longer than cooking or freezing, with some studies showing a curing period of at least 1 year, depending on the physicochemical parameters used [113]. Chilling meat also does not affect the viability of *T. gondii* tissue cysts [109]. In summary, freezing and cooking are the safest and most effective methods, while no standard concentrations and times are 100% effective in killing tissue cysts to advocate adopting and using these last methods [7].

## 6. Risk-based Control of Meat-borne Toxoplasmosis

Over ten years ago, EFSA received a mandate from the European Commission to evaluate meat inspection in a public health context aiming at the science-based modernisation of the process. Thus, EFSA performed a risk ranking of the principal biological hazards associated with meat from all domestic meat-producing animals based on the incidence and severity of human meat-borne diseases and proposed a comprehensive meat safety assurance system (MSAS) as the most effective approach to control the main hazards in the context of meat inspection [28,29,30,31,105,114,115]. This risk-based MSAS (RB-MSAS) focuses on high-risk (high-priority) public health hazards for which a meat safety risk reduction is envisaged by combining a range of preventive measures and controls applied both on the farm (pre-harvest phase) and at the abattoir (harvest phase) in a longitudinally integrated way. To this end, EFSA proposed harmonised epidemiological indicators (HEIs) for the risk categorisation of farms (presence of priority hazards in animal batches intended for slaughter) and abattoirs (capacity of reducing the relevant risk and setting appropriate targets for final chilled carcasses). Within this context, an epidemiological indicator is defined as “the prevalence or incidence of the hazard at a certain stage of the food chain or an indirect measure of the hazards that correlates to human health risk caused by the hazard”. The *T. gondii* public health relevance and proposed HEIs in livestock and poultry in the EU, according to EFSA, are summarised in Table 2.

Regarding classification in terms of priority, congenital or acquired toxoplasmosis incidence and severity are quite different (the first is rare but severe in contrast to the latter). However, due to available data indicating a high attribution of toxoplasmosis to the meat of pigs, small ruminants, and farmed boar and deer, *T. gondii* has been classified as a priority hazard in these animal species [31,105,114]. On the contrary, it could not be classified in terms of priority (undetermined) in the case of cattle and solipeds [30,115] due to the absence of robust epidemiological associations between their meat and toxoplasmosis, which induces high uncertainty in the relevant risk assessment.

As recently reviewed by Blagojevic et al. [116], the RB-MSAS in the EU, as proposed by EFSA, has not been practically materialised in its entity due to various practical challenges that remain to be resolved. For example, HEIs have not been introduced in a formalised way in any of the 18 European countries, including Greece, that participated in a recent study investigating the risk categorisation of abattoirs [117]. Moreover, no farm as yet has acquired the status of ‘controlled housing conditions’ in Greece, irrespective of the livestock species bred, though such a status is a component of the *T. gondii*-specific HEIs (Table 2). However, the high public health relevance of *T. gondii* in sheep and goat meat is particularly important from a Greek perspective, given the large number of small ruminants reared in the country and the various culinary traditions related to the consumption of their meat (carcasses and offal).

**Table 2:** *Toxoplasma gondii* public health relevance and proposed harmonised epidemiological indicators (HEIs) in the EU [28,29,30,31,105,114,115,118,119,120,121].

Animal Species	Public Health Relevance	Indicators (Animal/Food Category/Other)	Food Chain Stage	Analytical/Diagnostic Method	Specimen
Sheep and Goats	High	HEI 1: Farms with controlled husbandry conditions	Farm	Auditing	Not applicable
		HEI 2: Information on the age of the animals	Abattoir	Food chain information	Not applicable
		HEI 3: Detection of *T. gondii* infection	Abattoir	Serology	Blood
		HEI 4: Detection of *T. gondii* infection in older animals (more than one year) from farms with controlled husbandry conditions	Abattoir	Serology	Blood
		HEI 5: Absence of *T. gondii* infection in younger animals (less than one year) from farms without controlled husbandry conditions	Abattoir	Serology	Blood
Farmed deer and farmed wild boar	High	HEI 1: Detection of *T. gondii* antibodies in all farmed deer and wild boar	Abattoir	Serology	Meat juice
		HEI 2: Detection of *T. gondii* antibodies in the older animals (over one year) of farmed deer and wild boar	Abattoir	Serology	Meat juice
Swine	Medium	HEI 1: Farms with officially recognised controlled housing conditions (including control of cats and boots)	Farm	Auditing	Not applicable
		HEI 2: *T. gondii* in breeding pigs from officially recognised controlled housing conditions	Abattoir	Serology	Blood
		HEI 3: *T. gondii* in all pigs from non-officially recognised controlled housing conditions	Abattoir	Serology	Blood
Poultry (Broilers)	Low	Not applicable	Not applicable	Not applicable	Not applicable
Bovine	Undetermined	Not applicable	Not applicable	Not applicable	Not applicable

## 7. Conclusions

In Greece, sows, wild boars, hares, horses, and cats typically had lower seropositivity for *T. gondii* than the pooled European and worldwide values, but sheep and goats usually had greater seroprevalence compared to the European average values. No data were recorded on dairy products. There was substantial variation in studies of cattle and similar seroprevalence in chickens across Greece and Europe. Factors like feeding habits, housing conditions, and genetic predisposition influence infection risk among the different animal species, and control measures from consumers, such as freezing and thorough cooking of meat, are still necessary to reduce infection risk. Without any national mandatory screening of farm animals for antibodies and of carcasses for *T. gondii* tissue cysts, the infection risk for consumers is not under control. As regards meat-borne toxoplasmosis within the Greek context, the high public health relevance of *T. gondii* in sheep and goat meat (as per EFSA) is of particular interest. To this end, national initiatives towards the implementation of preventive measures and controls applied both on the small ruminant farms (pre-harvest phase) and at the abattoirs (harvest phase) in a longitudinally integrated way are necessary to effectively protect public health.

## Figures and Tables

**Table 1:** Studies on *Toxoplasma gondii* in animals in Greece.

A/A	Animal Species	Number of Animals Tested	Year of Publication	Location	Type of Study	Diagnostic Method	Results and Remarks
1	Pigs	609 sows (65 farms)	2016 [39]	Mainland Greece	Seroprevalence	IFAT and ELISA	4.3% (26/609). Risk factors: Farms in mountainous areas and farms with low biosecurity measures
2	Pigs	364 sows	2021 [38]	Not specified	Seroprevalence	IFAT	4.4% (16/364). Seropositive sows had higher AST and CK activity Risk factors: sows not vaccinated against porcine circovirus
3	Wild boars	94 wild boars	2015 [40]	Different areas of Greece	Seroprevalence	IFAT	5.2% (5/94)
4	Sheep and Goats	8700 sheep 2320 goats	1995 [48]	Crete	Seroprevalence	ELISA	Sheep: 23% (2001/8700) Goats: 14% (325/2320) Sheep had significantly higher seroprevalence than goats
5	Sheep	840 examined by IFAT 450 examined by ELISA	2001 [49]	Mainland Greece	Seroprevalence	IFAT and ELISA	IFAT: 53.4% (449/840) ELISA: 58.5% (263/450) All farms were conventional (non-organic)
6	Sheep and Goats	250 sheep (25 farms) 250 goats (26 farms)	2002 [46]	Southern Greece, Islands	Seroprevalence	IFAT	Sheep: 47.6% (119/250) Seroprevalence in abortive sheep: 52.1% (86/165) Goats: 50.4% (126/250) Seroprevalence in abortive goats:47.9% (69/144)
7	Sheep and Goats	182 sheep (9 farms) 167 goats (6 farms)	2007 [43]	Peloponnese, western Central Greece, and Ioannina	Seroprevalence	ELISA	Sheep: 50.5% (92/182) Seroprevalence in abortive sheep: 60.9% (14/23) Goats: 17.9% (30/167) Seroprevalence in abortive goats: 14.3% (7/49) All farms were organic Sheep had significantly higher seroprevalence than goats Sheep risk factors: Female sex, increased age Goat risk factors: Increased age
8	Sheep and Goats	289 sheep (37 farms) 174 goats (18 farms)	2009 [47]	Southern Greece	Seroprevalence	IFAT	Seroprevalence in abortive sheep: 49.8% (144/289) Seroprevalence in abortive goats: 29.9% (52/174) Sheep farms were semi-extensiveGoat farms were extensive
9	Sheep	500 sheep (1 farm)	2011 [50]	Northern Greece	Case Report	ELISA and histopathology	60% (300/500) of the sheep had aborted due to *T. gondii*
10	Sheep and Goats	1501 sheep (60 farms) 541 goats (41 farms)	2012 [41]	Northern Greece (Thessaloniki, Chalkidiki, Kastoria)	Seroprevalence	ELISA	Sheep: 48.6% (729/1501) Goats: 30.7% (166/541) No regional differences were found; sheep had significantly higher seroprevalence than goats Risk factors for both animal species: intensive or semi-intensive farming, feeding concentrate, water from public supply
11	Sheep and Goats	360 sheep (34 farms) 179 goats (20 farms)	2013 [45]	Thessaly	Seroprevalence	ELISA	Sheep: 28.3% (102/360) Goats: 16.8% (30/179) Risk factors for both animal species: herd size, anthelmintic treatment, class of anthelmintic, grazing with other flocks, farmer education, farm altitude, and generalised land cover
12	Sheep and Goats	458 sheep (50 farms) 375 goats (50 farms)	2013 [42]	Different areas of Greece	Seroprevalence	ELISA	Sheep: 53.7% (246/458). Goats: 61.3% (230/375) Goats had significantly higher seroprevalence than sheep
13	Goats	920 goats (3 farms)	2013 [51]	Northern Greece	Case Report	PCR, histopathology, serology	The abortion rate without treatment ranged from 11% to 78.5%
14	Sheep	80 sheep	2019 [44]	Trikala, Asimenio-Didimotiho, Xilokeriza-Corinthia, Velestino-Volos, Giannitsa, Sitihori-Didimotiho, Loutraki-Corinthia, Aliveri-Evia	Seroprevalence	MAT	56.25% (45/80). Risk factors: Geographic region, sheep from Trikala, Asimenio-Didimotiho, Xilokeriza-Corinthia Velestino-Volos, and Giannitsa, had significantly higher seroprevalence than sheep from Loutraki-Corinthia
15	Cattle	1890 cattle	1992 [53]	Serres	Seroprevalence	Complement fixation test	39.7% (751/1890)
16	Cattle	105 cattle	2005 [54]	Thessaloniki	Seroprevalence	ELISA	20% (21/105) well-managed intensive farms, Friesian cattle
17	Cattle	627 cattle(7 farms)	2020 [52]	Thessaly	Seroprevalence	ELISA	8.1% (51/627) All farms had previous reproductive problems, and cats present
18	Pigeons	379 domestic pigeons 50 wild pigeons	2011 [56]	Northern Greece	Seroprevalence	ELISA	Domestic pigeons 5.8% (22/379) Wild pigeons 0% (0/50)
19	Woodcock	86 woodcocks	2017 [55]	Macedonia, Mesolonghi	Molecular prevalence	PCR	4.7% (4/86)
20	Chickens	934 chickens (8 broiler farms, 14 backyard farms, 20 layer farms)	2022 [57]	Epirus, Central Macedonia, Central Greece-Attica	Seroprevalence	ELISA	9.4% (88/934) Risk factors: Farming system, nutrition type, and automatic feeding
21	Hares	105 hares	2019 [58]	Northern and Central Greece	Seroprevalence	IFAT	5.7% (6/105) No positive liver sample with PCR Risk factors: Precipitation indices and land uses
22	Equines	753 horses 13 mules 7 ponies	2010 [59]	Peloponnese, Attica, Thessaly, Macedonia	Seroprevalence	ELISA	1.8% (14/773) Risk factors: Activity type, location
23	Cats	1150 cats	2018 [61]	Countrywide	Faecal prevalence	Sedimentation and Flotation technique	0% (0/1150)
24	Cats	264 cats	2017 [62]	Crete	Faecal prevalence	Sedimentation and Flotation technique	0.4% (1/264) Oocysts were *T. gondii*-like, not confirmed with PCR
25	Cats	1554 cats	2022 [60]	Countrywide	Seroprevalence	Immunochromatographic test	21.8% (339/1554) Risk factors: hunting, rural areas, outdoor access
26	Wildcats	23 wildcat carcasses 62 faecal samples	2021 [63]	Different areas of Greece	Faecal prevalence	Sedimentation and Flotation technique	Faecal samples 1.6% (1/62) Faeces of necropsied animals 4.3% (1/23) Oocysts were *T. gondii*-like, not confirmed with PCR
27	Camel	1 Camel	[64]	Trikala	Case report	ELISA, PCR, cytology	The female camel was pregnant with a high antibody titer against *T. gondii* and aborted. The aborted foetus was positive for tissue cysts in brain smears and positive in PCR for *T. gondii*

Abbreviations: Aspartate aminotransferase (AST), creatine kinase (CK), enzyme-linked immunoassay (ELISA), indirect fluorescence antibody test (IFAT), modified agglutination test (MAT), polymerase chain reaction (PCR).

## Data Availability

Not applicable.
